# Identification and validation of biomarkers, construction of diagnostic models, and investigation of immunological infiltration characteristics for idiopathic frozen shoulder

**DOI:** 10.3389/fimmu.2025.1559422

**Published:** 2025-07-16

**Authors:** Han-tao Jiang, Li-ping Shen, Meng-Qi Pang, Min-jiao Wu, Jiang Li, Wei-jie Gong, Gang Jin, Rang-teng Zhu

**Affiliations:** ^1^ Department of Orthopedics, Taizhou Hospital of Zhejiang Province Affiliated to Wenzhou Medical, Taizhou, Zhejiang, China; ^2^ Department of Clinical Laboratory, Taizhou Hospital of Zhejiang Province Affiliated to Wenzhou Medical, Taizhou, Zhejiang, China; ^3^ Department of Operating Room, Taizhou Hospital of Zhejiang Province Affiliated to Wenzhou Medical, Taizhou, Zhejiang, China

**Keywords:** frozen shoulder, immune infiltration, transcriptomics, bioinformatics, nomogram

## Abstract

**Background:**

Idiopathic frozen shoulder (FS) can lead to difficulties in daily activities and significantly impact the quality of life. Early diagnosis and treatment can help alleviate symptoms and restore shoulder function. Therefore, we aimed to explore the diagnostic biomarkers and potential mechanisms of FS from a transcriptomics perspective.

**Methods:**

Total RNA was extracted from tissue samples of 15 FS and 11 controls. At the outset, we conducted differential expression analysis, weighted gene co-expression network analysis (WGCNA), and utilized the cytoHubba plugin, complemented by two machine learning algorithms, receiver operating characteristic (ROC) analysis, and expression level evaluation to identify biomarkers for FS. Subsequently, a nomogram was constructed based on the biomarkers. Additionally, we conducted enrichment and immune infiltration analyses to explore the mechanisms associated with these biomarkers. Finally, we confirmed the expression patterns of the biomarkers at the clinical level through reverse transcription-quantitative polymerase chain reaction (RT-qPCR).

**Results:**

*SNAI1*, *TWIST1*, *COL1A1*, *TUBB2B*, and *DCN* were identified as biomarkers for FS. The nomogram constructed based on them had a good predictive value for the occurrence of FS. Except for *DCN*, the other four genes were upregulated in FS samples, and the expression of *SNAI1*, *TWIST1*, and *TUBB2B* was also observed to be significantly upregulated in RT-qPCR. Moreover, these genes played important roles in pathways such as “ECM receptor interaction” and “lysosome”. We also found that the infiltration abundances of 11 types of immune cells were significantly upregulated in the FS samples, and they were positively correlated with each other. Our biomarkers showed strong correlations with these immune cells; *DCN* generally displayed a negative correlation, while the other four genes were generally positively correlated.

**Conclusion:**

This study established a link between FS biomarkers that have strong diagnostic potential and specific immune responses, highlighting possible targets for diagnosing and treating FS.

## 1 Introduction

Idiopathic frozen shoulder (FS), or adhesive capsulitis, is a painful, debilitating condition characterized by shoulder joint stiffness and functional impairment. It affects approximately 2-5% of the population, predominantly women and individuals aged 40 to 60 (1, 2). FS is typically self-limiting, with most individuals experiencing symptom resolution within two years (2). However, some individuals may endure persistent symptoms and functional limitations. Diagnosis relies primarily on clinical evaluation (3). In the absence of objective diagnostic criteria, particularly in the early stages, diagnosing FS can be challenging (4). FS progresses through three phases. The freezing phase involves increasing pain and stiffness. The stiffness phase maintains severe stiffness with reduced pain. The thawing phase gradually restores mobility over months. The entire process typically spans 1–3 years (5).

FS is classified as either primary (idiopathic) or secondary, with primary FS having an unknown etiology, while secondary FS can result from trauma, surgery, myocardial infarction, or conditions such as type I or type II diabetes, hypothyroidism, or Parkinson’s disease (6). Notably, Arkikila et al. (7) reported FS incidences of 10% in individuals with type I diabetes and 22% in those with type II diabetes.

The pathological characteristics of FS are thought to stem from a combination of inflammation and fibrosis (8). In the early stages of FS, inflammatory cells infiltrate the synovium, leading to synovial thickening and vascular hyperplasia (9–12). As the condition progresses, collagen deposition gradually increases in synovial tissues, resulting in joint capsule contracture and stiffness (10). During the inflammatory process, levels of pro-inflammatory cytokines (such as IL-1β and TNF-α) become elevated in synovial tissues (13, 14). These cytokines further exacerbate the inflammatory response and promote fibrotic processes (11). Concurrently, inflammatory stimuli induce fibroblast proliferation within the synovial tissues (15). Additionally, fibrosis of the shoulder joint capsule leads to restricted external rotation function, a phenomenon that has been clinically confirmed through dynamic ultrasound imaging (16). While inflammation is recognized as a key factor in FS development, the underlying causes and molecular mechanisms remain poorly understood (1). Current treatment modalities for FS include conservative therapies and surgical interventions (5). Although some improvement is observed in patients, long-term follow-up reveals that many still experience permanent functional disabilities (17). Therefore, further research into the pathogenesis of FS is crucial to enhancing treatment strategies.

Transcriptional research on FS has been limited. Evidence suggests a genetic predisposition to FS, as indicated by family history and racial predisposition (18). Analyzing gene expression changes could deepen our understanding of FS pathogenesis. For example, Cui et al. (8) used RNA sequencing to explore idiopathic FS pathogenesis by comparing tissue samples from five patients with FS to two individuals with acromioclavicular dislocations. Similarly, Hagiwara et al. (19) compared tissue samples from 12 patients with FS to 18 individuals with rotator cuff tears, identifying differentially expressed genes involved in fibrosis, inflammation, chondrogenesis, and angiogenesis. However, transcriptomic studies on FS remain scarce, with small sample sizes. Furthermore, the diagnostic value of biomarkers and their potential for drug prediction have not been sufficiently explored in current research.

A novel transcriptomic approach could provide valuable insights into FS pathogenesis and lead to the identification of new biomarkers for diagnosis and treatment. This study investigates potential diagnostic biomarkers for FS and their molecular regulatory mechanisms through bioinformatics analysis of shoulder joint tissues from patients with FS and healthy controls. The findings offer new perspectives on the clinical diagnosis, prevention, and management of FS.

## 2 Materials and methods

### 2.1 Sample and date collection

Biopsies were obtained from the rotator interval of the glenohumeral joint capsule in both control individuals and patients with FS. Control samples were collected from 11 individuals undergoing elective shoulder stabilization surgeries, while FS samples came from 15 patients diagnosed with Stiffness-Stage FS, defined by at least 6 months of symptoms, who underwent arthroscopic capsular release (**Supplementary Table 1**). Patients with potential secondary causes of FS, including shoulder trauma, postoperative shoulder conditions, cerebral infarction, postoperative breast cancer, and diabetes, were excluded from the study. No statistically significant differences in age or gender were observed between the two groups. The relevant transcriptomic datasets of rheumatoid arthritis (RA) (GSE55235) and rotator cuff tears (RCT) (GSE199484) were obtained from the GEO database to verify the expression specificity of biomarkers in FS. The 20 control samples and 33 RA samples from GSE55235 (GPL96) were selected for analysis. Five disease samples and five control samples were selected from GSE199484 (GPL24676) for analysis.

### 2.2 RNA sequencing and data preprocessing

Total RNA was extracted from the tissue samples of 15 FS and 11 controls using TRIzol (Invitrogen, CA, USA). RNA quality and quantity were assessed with a NanoDrop ND-1000 spectrophotometer (Wilmington, DE, USA) and a Bioanalyzer 2100 system (Agilent, CA, USA), respectively. RNA was then used for library construction with the Hieff NGS Ultima Dual-mode mRNA Library Prep Kit for Illumina, aiming to generate libraries with a target fragment size of 300 bp ± 50 bp. Sequencing was performed on the Illumina NovaSeq 6000 platform using the bipartite PE150 sequencing mode. The distribution of base types was analyzed to check for AT/GC separation. Using the human gene annotation file GRCh38 (hg38) (20) as the reference gene set, the data was processed to generate COUNT data (**Supplementary Table 2**).

### 2.3 Selection of differentially expressed genes and key module genes

For differential expression analysis between FS and control samples, the DESeq2 package (v 1.38.0) (21) was employed, with the criteria for DEGs set as |log_2_Fold Change (FC)| > 1 and adj.*P* < 0.05. The ggplot2 package (v 3.4.4 (22) and pheatmap package (v 1.0.12) (23) were used to create volcano plot and heatmap, respectively, illustrating the top 10 upregulated and downregulated genes ranked by log_2_FC.

Next, the WGCNA package (v 1.71) (24) was used to construct a co-expression network with FS as the trait to identify key module genes. Hierarchical clustering based on the Euclidean distance of expression levels was conducted to identify and remove outliers from the dataset. The scale-free fit index (R^2^) was set to exceed 0.85, while the mean connectivity approached zero, ensuring the optimal soft threshold power (β) for a scale-free gene network. Genes were then partitioned into modules using the dynamic tree cut algorithm, with a cutree parameter of 4 and a module merging threshold of 0.25, ensuring that each module contained a minimum of 200 genes. The ssGSEA algorithm from the GSVA package (v 1.46.0) (24) was utilized to compute scores for each FS sample. The correlation between the modules and FS scores was analyzed using the Pearson correlation function, and a heatmap was generated to visualize these correlations. The modules with the highest positive and negative correlations to FS scores were selected as key modules, and the genes within these modules were defined as key module genes.

### 2.4 Enrichment analysis and protein-protein interaction network construction

To identify candidate genes, the intersection of DEGs and key module genes was analyzed using the ggvenn package (version 0.1.9) (25). Subsequently, Gene Ontology (GO) and Kyoto Encyclopedia of Genes and Genomes (KEGG) enrichment analyses were performed on these candidate genes using the clusterProfiler package (version 4.7.1.003) (26), with a significance threshold of *P* < 0.05. The GO analysis covered biological processes, molecular functions, and cellular components. Candidate genes were entered into the STRING database (http://string-db.org) with an interaction score threshold of 0.9. The resulting PPI network was visualized using Cytoscape software (version 3.9.1) (27). The cytoHubba plugin was used to identify hub genes based on Betweenness centrality values, selecting the top 10 genes for further analysis.

### 2.5 Identification of candidate biomarkers through machine learning algorithm

Following the identification of hub genes, two machine learning algorithms were employed to screen for candidate biomarkers. The glmnet package (v 4.1-8) (28) was employed to perform LASSO regression, selecting genes with non-zero regression coefficients at the minimum lambda value. Additionally, the Boruta algorithm, implemented *via* the Boruta package (v 8.0.0) (29), was used to select genes based on their importance scores. The intersecting genes from both algorithms were then defined as candidate biomarkers.

### 2.6 Assessment of diagnostic performance of biomarkers

The candidate biomarkers were assessed using the pROC package (version 1.18.5) to generate ROC curves, evaluating their ability to differentiate between FS and control samples, and calculating the area under the curve (AUC). Additionally, the expression levels of the candidate biomarkers in both FS and control samples were analyzed. Genes meeting two criteria—AUC > 0.7 and significant differential expression (*P* < 0.05) between FS and control samples—were identified as biomarkers.

Next, the biomarkers were integrated into a nomogram using the rms package (v 6.7-1) (30), optimizing the diagnostic value of these biomarkers. In the nomogram, each biomarker was assigned a score, and the total score was the sum of the individual scores. The total score could then be used to estimate the incidence rate of FS, with higher scores indicating greater disease risk. To evaluate the predictive accuracy and performance of the nomogram, both the calibration curve and ROC curve were plotted separately.

### 2.7 Functional and annotation analysis

Spearman correlation analysis was performed using the psych package (v 2.4.1) (31) to evaluate correlations among the biomarkers. The correlation heatmap was created using the corrplot package (v 0.92) (32), with thresholds set to |cor| > 0.3 and *P* < 0.05. Furthermore, the c2.cp.kegg.v7.4.symbols.gmt file from the Molecular Signatures Database (MSigDB) (https://www.gsea-msigdb.org/gsea/msigdb) was downloaded as the background gene set. The correlation between each biomarker and other genes in the transcriptome dataset was computed. GSEA was performed using the clusterProfiler package to explore the signaling pathways associated with the biomarkers (*P* < 0.05). The enrichplot package (v 1.18.4) (32) was utilized to visualize the top 5 pathways linked to each biomarker.

### 2.8 Immune infiltration analysis

To assess immune microenvironment differences between FS and healthy individuals, the ssGSEA algorithm from the GSVA package was used to calculate immune cell scores for 28 immune cell types (33) in each sample. These scores were then compared between FS and control samples (*P* < 0.05). Additionally, Spearman correlation analysis was performed to explore the relationships among differential immune cells and their associations with biomarkers, with a threshold set to |cor| > 0.3 and *P* < 0.05.

### 2.9 Construction of regulatory network and drug prediction analysis

The regulatory mechanisms of the biomarkers were investigated. The multiMiR package (v 1.20.0) (33) was used to predict upstream miRNAs of the biomarkers from the miRDB (http://www.mirdb.org/) and TargetScan databases (https://www.targetscan.org/vert_80/). The intersection of gene-miRNA relationships from these two databases was analyzed to obtain the final set of miRNAs. The starBase database was then employed to predict upstream lncRNAs associated with these miRNAs. A lncRNA-miRNA-mRNA regulatory network was constructed using Cytoscape software. Additionally, transcription factors (TFs) targeting the biomarkers were identified from the hTFtarget database (https://guolab.wchscu.cn/hTFtarget/#!/). The previously identified miRNAs were used to map the TF-mRNA-miRNA regulatory network in Cytoscape software. Furthermore, to explore potential therapeutic drugs for FS, the biomarkers were queried in the DGIDB (https://dgidb.org), and the predicted drugs were imported into Cytoscape to visualize the drug-biomarker network.

### 2.10 Expression validation of biomarkers

Tissue samples were collected from 15 patients with FS and 11 healthy controls at the Ethics Committee of Taizhou Hospital in Zhejiang Province. All samples were analyzed *via* reverse transcription-quantitative polymerase chain reaction (RT-qPCR). The study received approval from the Ethics Committee of Taizhou Hospital (K20210708). To confirm biomarker expression, total RNA was extracted from 10 tissue samples using TRIzol (Ambion, Austin, USA), following the manufacturer’s instructions. Reverse transcription was performed using the SureScript First-Strand cDNA Synthesis Kit (Servicebio, Wuhan, China). RT-qPCR was carried out with the 2xUniversal Blue SYBR Green qPCR Master Mix (Servicebio, Wuhan, China). The primer sequences used for PCR are provided in **Supplementary Table 3**, with GAPDH as the internal reference gene. Gene expression levels were determined using the 2^−ΔΔCt^ method (34).

### 2.11 Specificity verification of biomarkers

In order to investigate whether the expression changes of biomarkers were specific in FS, the Wilcoxon test was used to analyze the differences in the expression levels of biomarkers between the disease samples and control samples in RA in GSE55235 dataset, and RCT in GSE199484 dataset, (*P* < 0.05).

### 2.12 Statistical analysis

All analyses were performed using R software (v 4.2.2). Group differences were assessed using the Wilcoxon test, and a *P*-value of less than 0.05 was considered statistically significant.

## 3 Results

### 3.1 Identification of 2,136 DEGs and 4,800 key module genes

**Figure 1:**
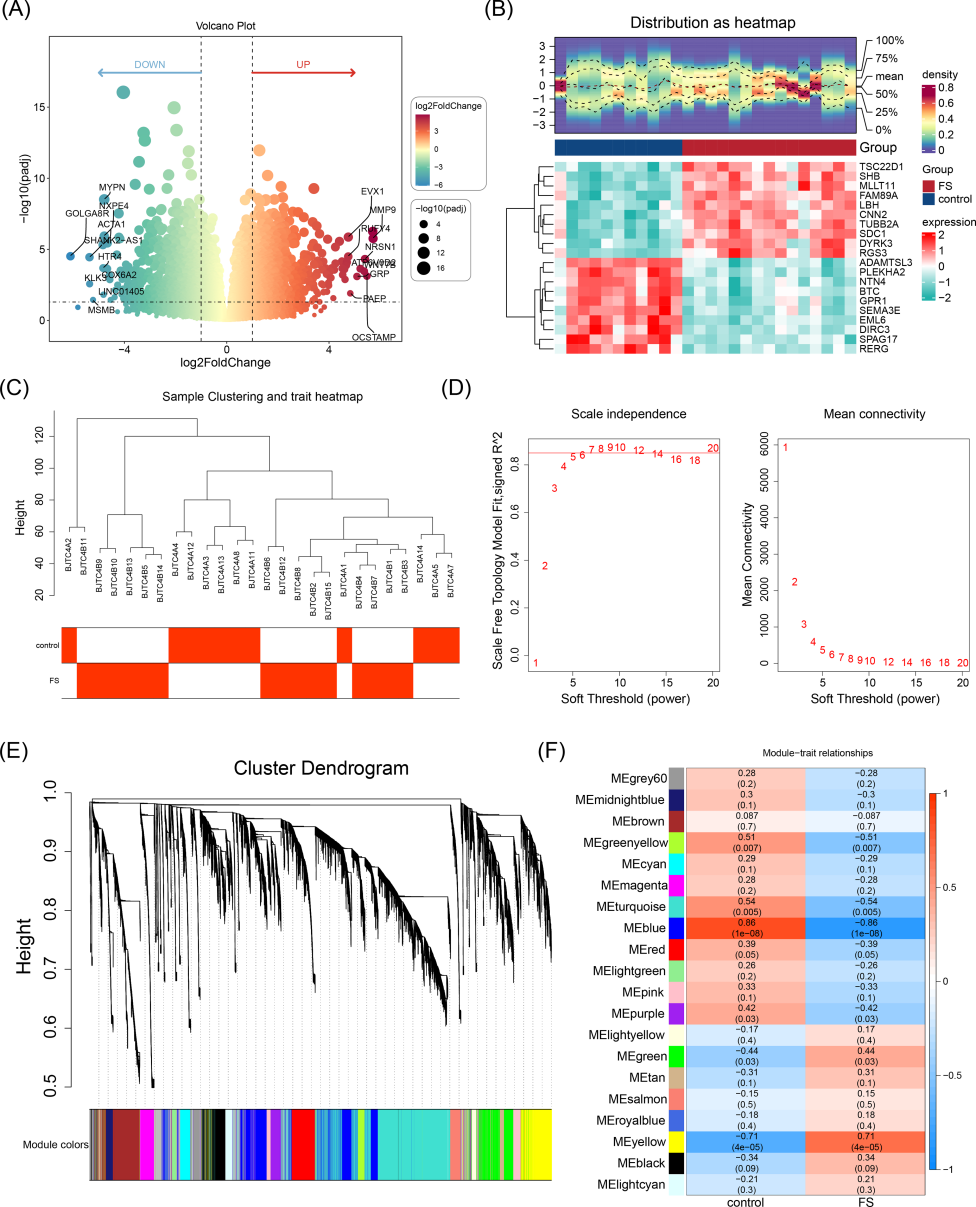
Identification of DEGs and key module genes. **(A)** Volcano plot of differentially expressed genes. Red indicates upregulated genes, and green indicates downregulated genes. Each dot represents a gene. The genes marked in the volcano map were the top 10 up-regulated genes and the top 10 down-regulated genes sorted by log_2_FC. **(B)** Heatmap of differentially expressed genes. The blue color indicated low expression, and the red color indicated high expression. The bluer the blue color was, the lower the expression was, and the redder the red color was, the higher the expression was. **(C)** Hierarchical clustering analysis. **(D)** Selection of the optimal soft threshold power value. The optimal soft threshold power was 9. **(D-1)** The left panel shows the scale-free model fit index. **(D-2)** The right panel shows the mean connectivity of these values. **(E)** Cluster dendrogram of genes enriched based on dissimilarity measure and assigned modules. Clustered into 20 co-expression modules. **(F)** Heatmap showing the correlation between module genes and FS. The blue color represented a negative correlation, and the red color represented a positive correlation. The darker the color, the stronger the correlation.

Differential expression analysis identified 2,136 DEGs between FS and control samples, with 964 upregulated and 1,172 downregulated genes (**Figures 1A, B**). FS was then used as a trait to construct a co-expression network, and clustering analysis revealed no outliers, allowing the inclusion of all samples in subsequent analyses (**Figure 1C**). The optimal soft threshold power (β) for constructing a scale-free network was determined to be 9, where the scale-free fit index (R^2^) reached 0.8730 and mean connectivity approached zero (**Figure 1D**). Using a cutree parameter of 4, a module merging threshold of 0.25, and a minimum of 200 genes per module, 20 co-expression modules were identified (**Figure 1E**). A correlation heatmap showed that the MEblue module had the strongest negative correlation with FS scores (cor = -0.86, *P* < 0.05), while the MEyellow module exhibited the strongest positive correlation (cor = 0.71, *P* < 0.05), designating them as key modules (**Figure 1F**). These two modules collectively contained 4,800 key module genes.

### 3.2 Identification of 10 hub genes with crucial roles in network information transmission

**Figure 2:**
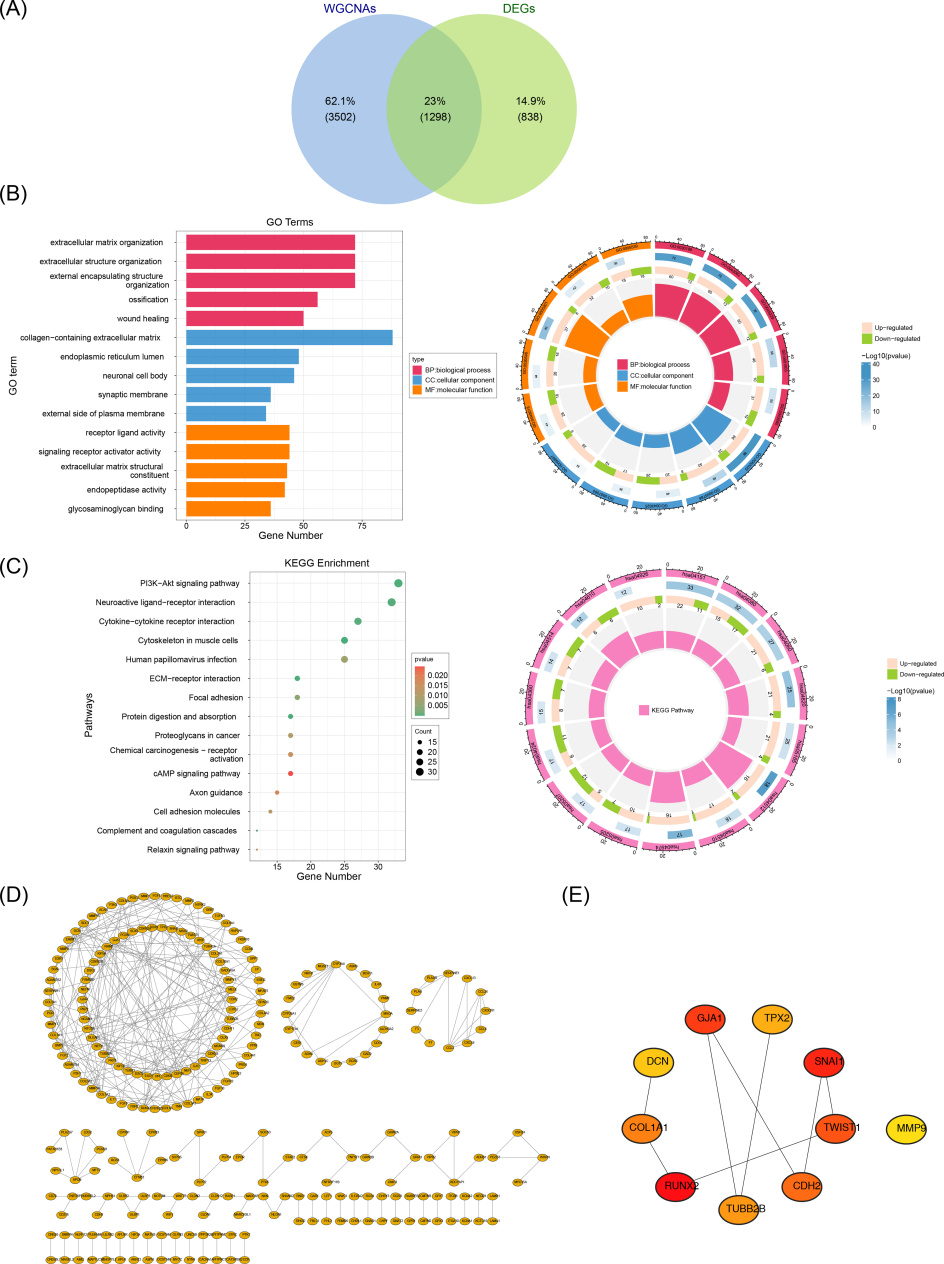
Acquisition and enrichment analysis of candidate genes. A total of 1,298 candidate genes were screened out. **(A)** Identification of candidate genes. **(B)** GO enrichment analysis of candidate genes. **(C)** KEGG enrichment analysis of candidate genes. **(D)** PPI network of candidate genes. **(E)** Identification of hub genes. A total of 10 hub genes were screened out.

Intersecting the 2,136 DEGs with the 4,800 key module genes yielded 1,298 candidate genes (**Figure 2A**). To explore the biological functions and pathways involved in the candidate genes, GO and KEGG analyses were performed. Enrichment analysis of these genes revealed 977 associated GO terms, including 783 BP, 89 CC, and 105 MF, with a focus on extracellular matrix organization, collagen-rich extracellular matrix, and endoplasmic reticulum (**Figure 2B**). Additionally, 41 KEGG pathways were linked to the PI3K-Akt signaling pathway, neuroactive ligand-receptor interactions, and cytokine-cytokine receptor interactions, among others (**Figure 2C**). These results provide further insight into the roles of these pathways in FS pathogenesis.

We constructed a PPI network using these candidate genes to explore their interaction relationships at the protein level. The PPI network comprised 257 nodes and excluded 1,041 outlier genes. The network contained 300 edges, an average node degree of 0.621, an average local clustering coefficient of 0.188, and a PPI enrichment *P*-value of < 1 × 10^-16^ (**Figure 2D**). The cytoHubba plugin identified 10 hub genes based on Betweenness values: RUNX2, *SNAI1*, GJA1, *TWIST1*, CDH2, *COL1A1*, *TUBB2B*, TPX2, *DCN*, and MMP9 (**Figure 2E**).

### 3.3 Selection of *SNAI1*, *TWIST1*, *COL1A1*, *TUBB2B*, and *DCN* as candidate biomarkers

**Figure 3:**
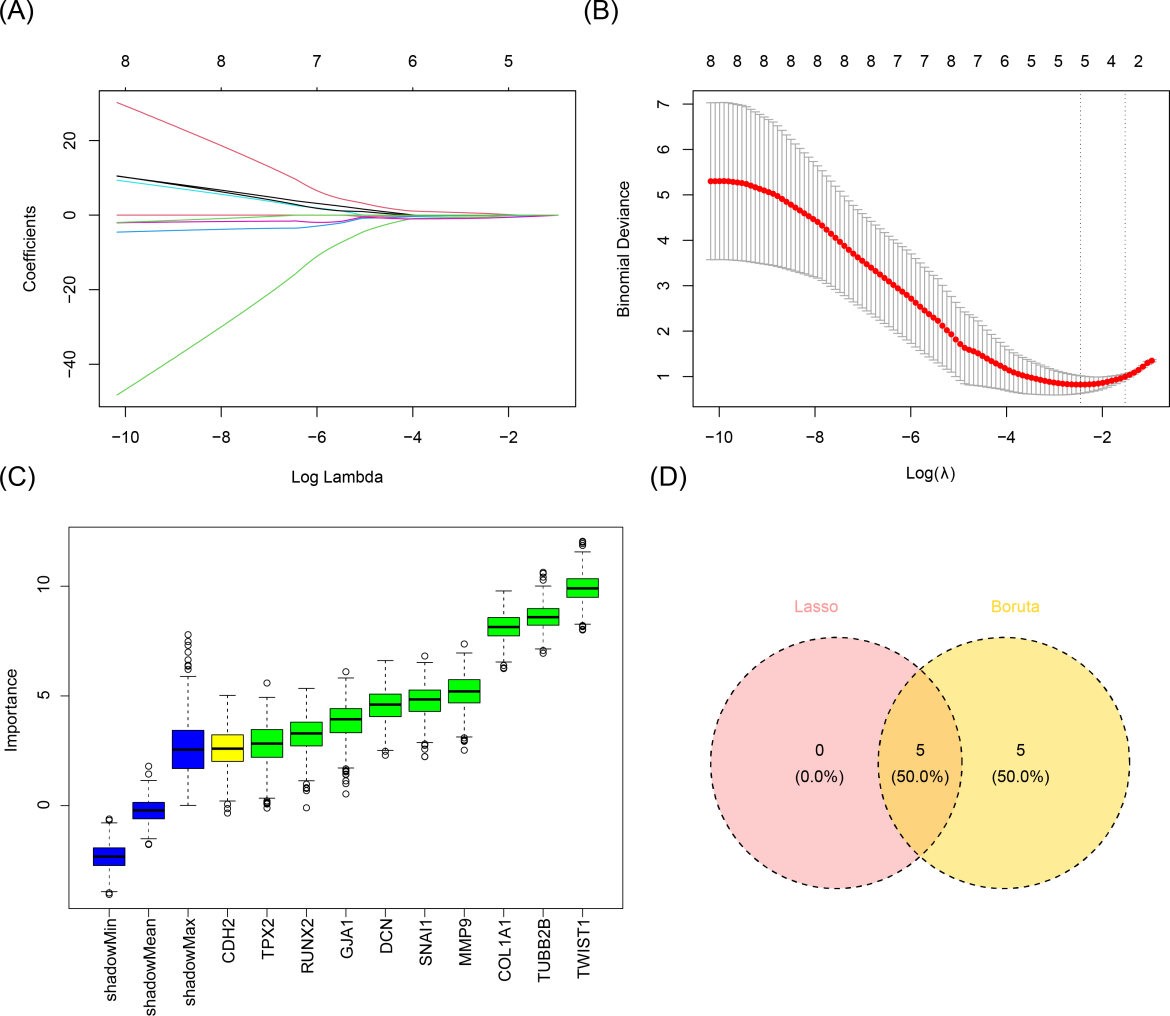
Acquisition of candidate biomarkers. **(A, B)** Results of the LASSO analysis. Five genes were screened when lambda.min was equal to 0.08578354 and log(lambda) was equal to -2.455928. **(C)** Results of the Boruta method analysis. Ten genes were screened out. **(D)** Identification of candidate biomarkers. A total of five candidate biomarkers were obtained.

In order to further screen the candidate biomarkers, we performed the LASSO and Boruta analyses. LASSO analysis identified five genes when lambda.min = 0.08578354 and log(lambda) = -2.455928: *SNAI1*, *TWIST1*, *COL1A1*, *TUBB2B*, and *DCN* (**Figures 3A, B**). Concurrently, the Boruta method identified 10 genes: RUNX2, *SNAI1*, GJA1, *TWIST1*, CDH2, *COL1A1*, *TUBB2B*, TPX2, *DCN*, and MMP9 (**Figure 3C**). Overlapping the results of these machine learning algorithms revealed five candidate biomarkers: *SNAI1*, *TWIST1*, *COL1A1*, *TUBB2B*, and *DCN* (**Figure 3D**).

### 3.4 Constructing a nomogram with robust diagnostic performance using biomarkers

**Figure 4:**
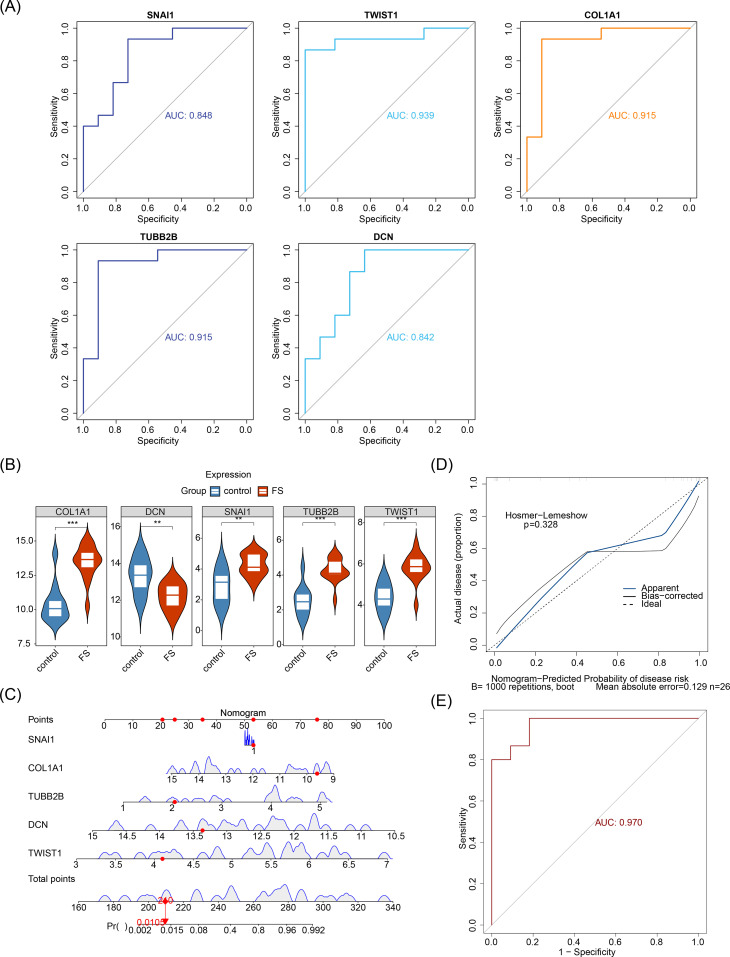
Prognostic analysis of biomarkers. **(A)** ROC analysis of candidate biomarkers. **(B)** Expression analysis of candidate biomarkers. “**” represents *P* < 0.01, and “***” represents *P* < 0.001. **(C)** Construction of the nomogram. **(D)** Calibration curve for the nomogram. **(E)** ROC analysis of the nomogram.

To screen out the biomarkers, ROC analysis and expression level analysis were conducted on the candidate biomarkers. ROC analysis demonstrated that the AUC values for *SNAI1*, *TWIST1*, *COL1A1*, *TUBB2B*, and *DCN* were 0.845, 0.939, 0.915, 0.915, and 0.842, respectively, all surpassing the 0.7 threshold (**Figure 4A**), indicating their robust ability to differentiate FS from normal samples and suggesting potential diagnostic value. Expression analysis revealed significant differences in the expression of all five biomarkers between FS and control samples (*P* < 0.05) (**Figure 4B**). With the exception of *DCN*, the other four genes were upregulated in FS samples (*P* < 0.05). Consequently, *SNAI1*, *TWIST1*, *COL1A1*, *TUBB2B*, and *DCN* were identified as key biomarkers in this study.

Furthermore, to explore the predictive ability of biomarkers for the occurrence probability of FS, these biomarkers were incorporated into a nomogram, with individual scores assigned to each (**Figure 4C**). The total score predicted the FS incidence rate, with higher scores indicating greater disease risk. The calibration curve closely aligned with the threshold line, demonstrating excellent predictive accuracy for the nomogram (*P* = 0.328) (**Figure 4D**). ROC analysis revealed that the overall AUC for the nomogram was 0.970, significantly surpassing that of any single gene (**Figure 4E**), further validating the nomogram’s strong diagnostic capability for FS.

### 3.5 Exploring the pathways by which biomarkers influence FS development

**Figure 5:**
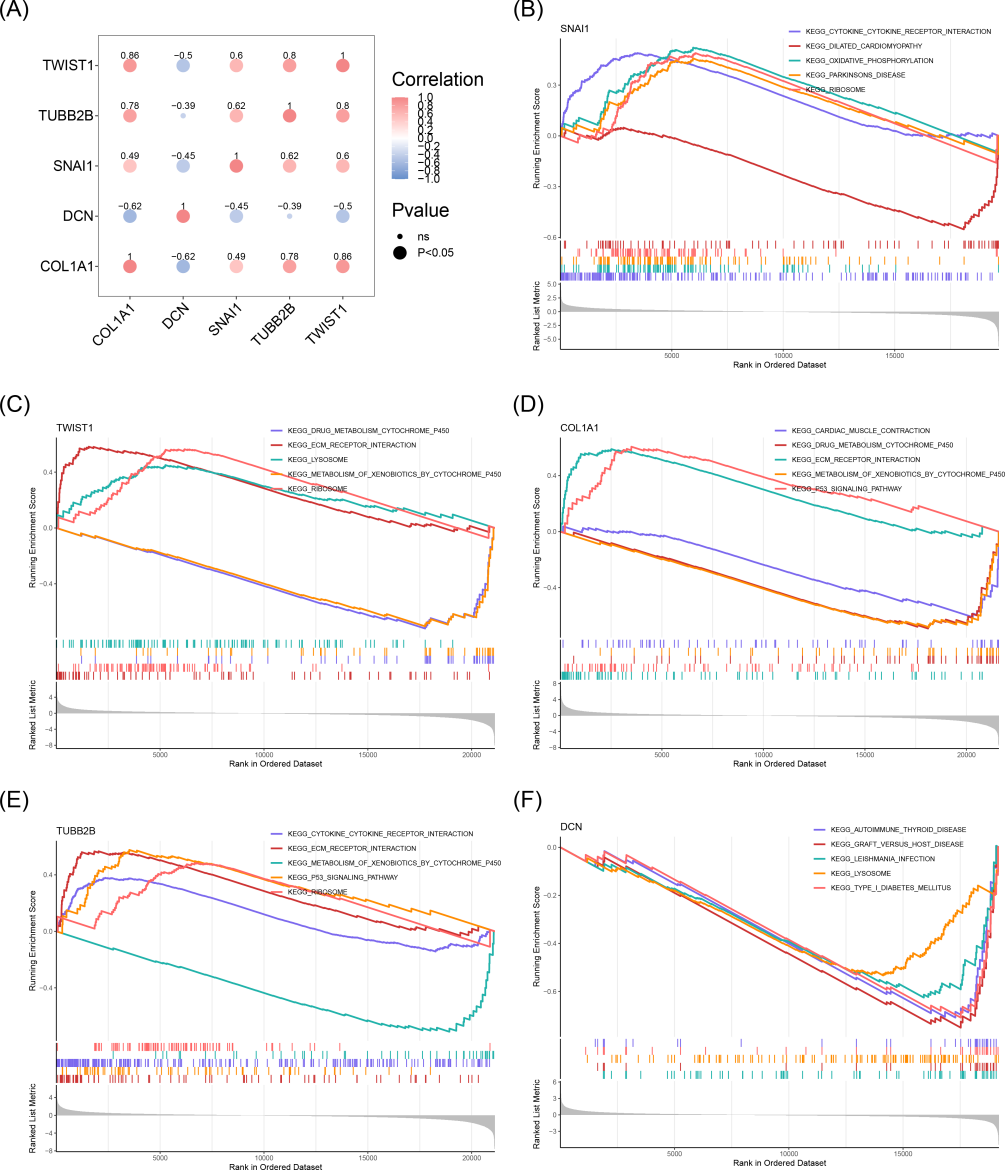
Results of GSEA enrichment analysis of biomarkers. **(A)** Correlation between biomarkers. The blue color represented a negative correlation, and the red color represented a positive correlation. The darker the color, the stronger the correlation. **(B)** GSEA enrichment analysis results for *SNAI1*. **(C)** GSEA enrichment analysis results for *TWIST1*. **(D)** GSEA enrichment analysis results for *COL1A1*. **(E)** GSEA enrichment analysis results for *TUBB2B*. **(F)** GSEA enrichment analysis results for *DCN*.

In order to evaluate the correlations among biomarkers, Spearman’s correlation analysis was performed. Except for *DCN* and *TUBB2B*, all biomarker pairs showed moderate to strong correlations (|cor| > 0.3, *P* < 0.05). The strongest positive correlation was between *COL1A1* and *TWIST1* (cor = 0.86), while the most substantial negative correlation was between *COL1A1* and *DCN* (cor = -0.62) (**Figure 5A**). GSEA identified several biological mechanisms and signaling pathways associated with the biomarkers, including “ribosome”, “lysosome”, “drug metabolism-cytochrome P450”, “ECM receptor interaction”, “metabolism of xenobiotics by cytochrome P450”, and “p53 signaling pathway” (**Figures 5B-F**). These pathways encompass key biological processes such as protein synthesis in the ribosome, waste degradation in lysosomes, cytochrome P450-mediated drug and xenobiotic metabolism, ECM-cell interactions, and cellular stress responses *via* the p53 signaling pathway. Together, these interconnected processes form a complex network essential for maintaining cellular function and sustaining life.

### 3.6 Enhanced immune cell infiltration and gene expression correlations in FS reveal potential therapeutic targets

**Figure 6:**
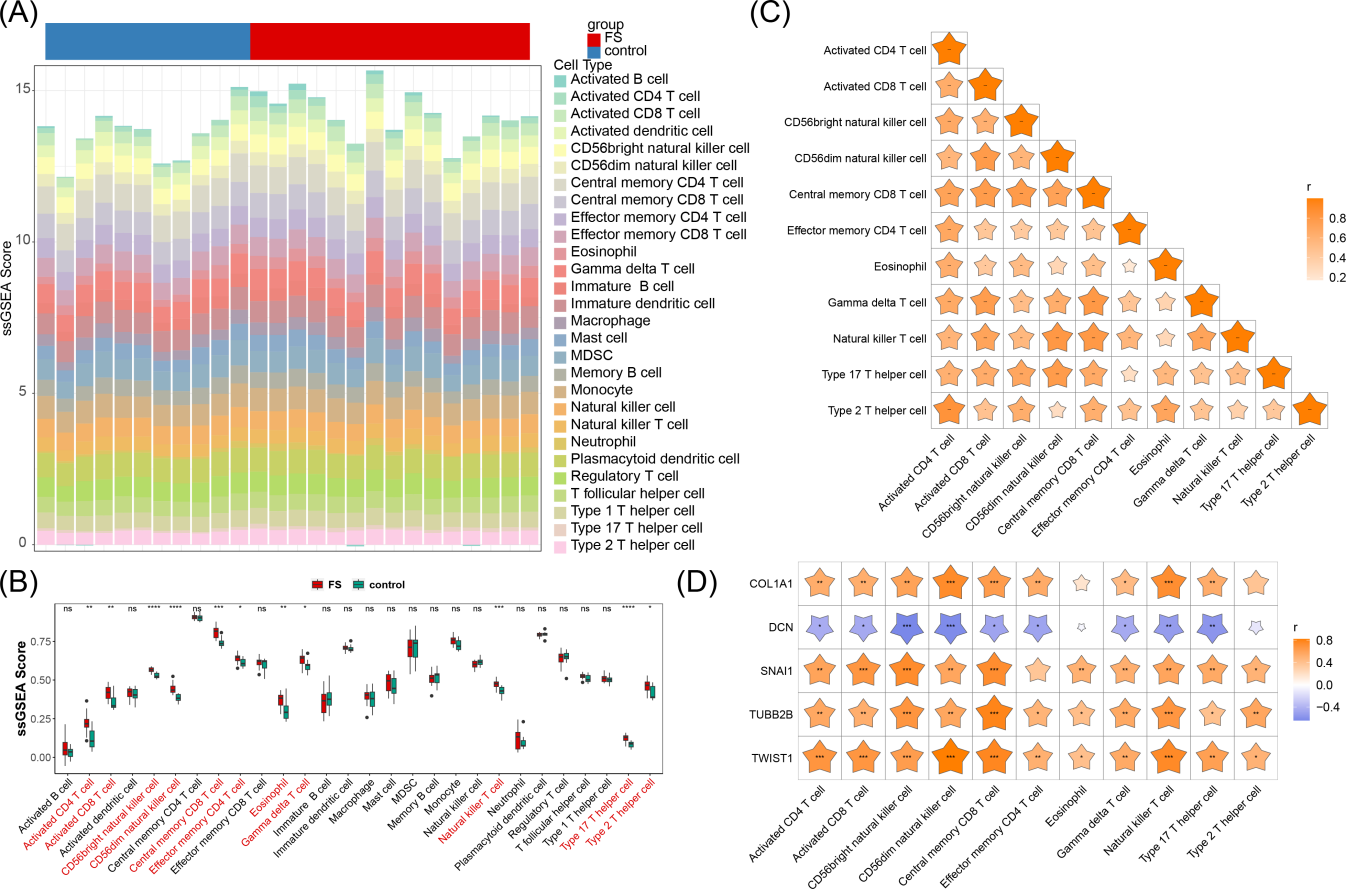
Immune cell infiltration. **(A)** Abundance of 28 immune cell infiltrates in FS and control samples. **(B)** Differences in immune cell infiltration between FS and control samples. “ns” represents *P* > 0.05, “*” represents *P* < 0.05, “**” represents *P* < 0.01, “***” represents *P* < 0.001, and “****” represents *P* < 0.0001. **(C)** Correlation between differential immune cells. “*” represents *P* < 0.05, “**” represents *P* < 0.01, “***” represents *P* < 0.001. **(D)** Correlation between biomarkers and differential immune cells. “*” represents *P* < 0.05, “**” represents *P* < 0.01, “***” represents *P* < 0.001.

To explore the differences in immune cell infiltration between the FS samples and the control samples, an immune infiltration analysis was performed. Our analysis employed a stacked bar chart to illustrate the abundance of 28 immune cell infiltrates in FS and control samples (**Figure 6A**), while a box plot further emphasized the significant differences in these immune cells between the two groups (*P* < 0.05) (**Figure 6B**). Eleven immune cell types exhibited significant differences, including activated CD4 T cells, activated CD8 T cells, CD56bright natural killer cells, CD56dim natural killer cells, central memory CD8 T cells, effector memory CD4 T cells, eosinophils, gamma delta T cells, natural killer T cells, type 17 T helper cells, and type 2 T helper cells. Notably, all 11 immune cell types showed higher infiltration in FS samples (*P* < 0.05), suggesting their potential involvement in the pathophysiology of FS.

Further analysis revealed that these differentially infiltrating immune cells displayed varying degrees of positive correlation with one another (**Figure 6C**). Type 2 T helper cells exhibited the strongest positive correlation with activated CD4 T cells (cor = 0.85, *P* < 0.05), suggesting a possible synergistic role in FS immune responses, potentially contributing to inflammation or immune regulation.

Additionally, *DCN* generally demonstrated a negative correlation with these immune cells, while the other four genes (*SNAI1*, *TWIST1*, *COL1A1*, and *TUBB2B*) were positively correlated with these immune cells, particularly *SNAI1*, *TWIST1*, and *TUBB2B* (cor > 0.3, *P* < 0.05) (**Figure 6D**). Further investigation into the roles of these immune cells and the modulation of associated gene expression and signaling pathways could offer new approaches for mitigating FS symptoms and progression.

### 3.7 Unraveling regulatory networks and drug studies

**Figure 7:**
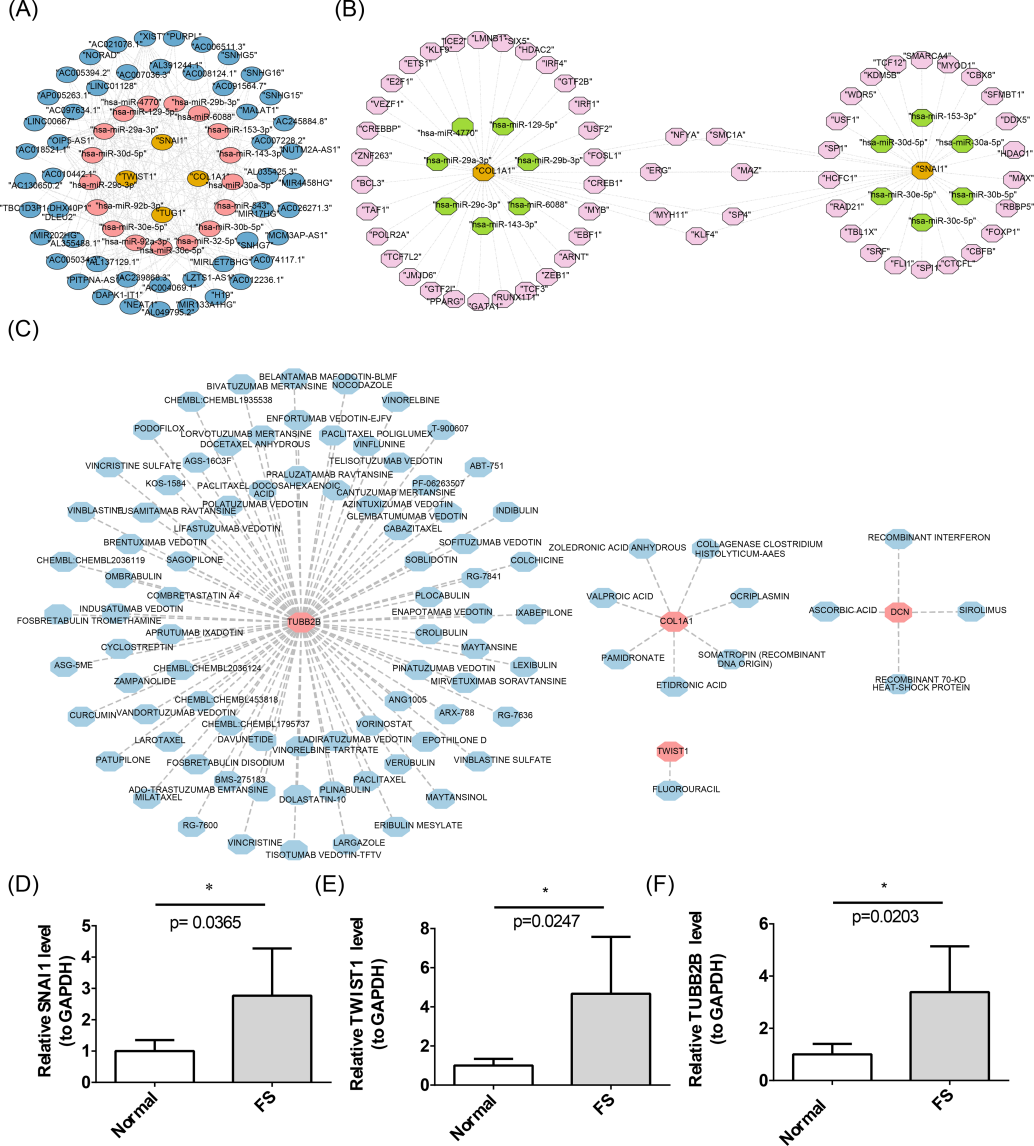
Construction of molecular networks. **(A)** The lncRNA-miRNA-mRNA network. The yellow color represents biomarkers, the pink color represents target miRNAs, and the blue color represents lncRNAs. **(B)** Construction of the TF-mRNA-miRNA network. In the figure, the yellow color represents biomarkers, the pink color represents TFs, and the green color represents miRNAs. **(C)** Networks of biomarkers and targeted drugs. The pink color represents biomarkers, and the blue color represents drugs. **(D-F)** Results of RT-qPCR analysis of biomarkers. The error bars in the figure represent the standard deviation. “*” represents *P* < 0.05.

For the purpose of understanding the miRNAs, lncRNAs, TFs, and drugs targeting the biomarkers, the construction of a molecular regulatory network and drug prediction were carried out. A total of 146 miRNAs were predicted using miRDB, and 93 miRNAs were identified through TargetScan, resulting in an intersection of 17 miRNAs. Subsequently, 50 upstream lncRNAs for these 17 miRNAs were predicted *via* starBase. This data facilitated the construction of a lncRNA-miRNA-mRNA network encompassing four biomarkers (*SNAI1*, *TWIST1*, *COL1A1*, *TUBB2B*), 17 miRNAs, and 50 lncRNAs, revealing complex interaction dynamics. For example, AC005034.3 may regulate TUG1 *via* hsa-miR-30e-5p (**Figure 7A**). Using hTFtarget, 37 TFs targeting *COL1A1* and 29 targeting *SNAI1* were identified, enabling the creation of a TF-mRNA-miRNA network (**Figure 7B**). Notably, TFs such as ERG, NFYA, SMC1A, MAZ, MYH11, SP4, and KLF4 were predicted to regulate both *SNAI1* and *COL1A1*. In addition, based on DGIdb, one drug (FLUOROURACIL) was identified for *TWIST1*, seven drugs (e.g., VALPROIC ACID, PAMIDRONATE) for *COL1A1*, four drugs (e.g., ASCORBIC ACID, SIROLIMUS) for *DCN*, and 82 drugs (e.g., SAGOPILONE, SOBLIDOTIN) for *TUBB2B* (**Figure 7C**). No interacting drugs were predicted for *SNAI1*.

### 3.8 Verification of biomarkers expression

Previous studies revealed significantly higher expression levels of *SNAI1*, *TWIST1*, *COL1A1*, and *TUBB2B* in FS samples (*P* < 0.05), while *DCN* showed significantly lower expression (*P* < 0.05) (**Figure 4B**). To validate these findings, RT-qPCR was performed to measure gene expression in patients with FS. RT-qPCR results confirmed that SNAI1, TWIST1, and TUBB2B were expressed at significantly higher levels in FS samples (*P* < 0.05), consistent with prior results (**Figures 7D-F**).

### 3.9 The expression of biomarkers is specific in FS

To explore the specificity of biomarker expression changes in FS, we carried out validation analyses using the datasets from RA and RCT. The results showed that in RA, there were no significant differences in the expressions of *TUBB2B* and *DCN* between the RA samples and the control samples. *SNAI1* and *TWIST1* were significantly under-expressed in the RA samples (**Supplementary Figures 1A**), which was contrary to their expression trends in FS. In RCT, the expressions of the five biomarkers did not show any significance between the RCT samples and the control samples (**Supplementary Figures 1A**). This indicated that the expressions of these five biomarkers were specific in FS.

## 4 Discussion

Diagnosing early FS based solely on patient symptoms presents significant challenges, often leading to delays in both diagnosis and treatment due to the unclear pathogenesis of the condition (35). This study aimed to identify diagnostic biomarkers and explore the underlying mechanisms of FS from a transcriptomic perspective. The study identified *SNAI1*, *TWIST1*, *COL1A1*, *TUBB2B*, and *DCN* as biomarkers for FS, each demonstrating strong diagnostic potential. With the exception of *DCN*, the other four genes were upregulated in FS samples, a finding confirmed by RT-qPCR. These genes were involved in various biological processes and cellular mechanisms, being enriched in pathways such as “ECM receptor interaction”, “lysosome”, and “p53 signaling pathway”. Furthermore, 11 immune cell types showed significantly different infiltration abundances between FS and control samples, all of which were upregulated in FS samples. This study highlights the association between FS biomarkers with robust diagnostic value and specific immune responses, providing potential targets for FS diagnosis and intervention.

WGCNA, LASSO, and Boruta are powerful bioinformatics and statistical methods for identifying key genes or biomarkers from complex datasets (28, 36, 37). Each method offers unique advantages for different aspects of data analysis. WGCNA excels in identifying co-expression modules and linking them to external traits, offering a network-based perspective on gene interactions (36). LASSO is particularly useful for variable selection and regularization, making it suitable for high-dimensional data and predictive modeling (28). Boruta provides comprehensive feature selection by identifying all relevant features and is robust against overfitting and false positives (37). In summary, these methods effectively screen and identify biomarkers.

This study identified five biomarkers for FS: *SNAI1*, *TWIST1*, *COL1A1, TUBB2B*, and *DCN*. *SNAI1*, a transcription factor, plays a pivotal role in epithelial-mesenchymal transition (EMT), a process in which epithelial cells acquire mesenchymal, fibroblast-like properties, such as enhanced migratory ability and resistance to apoptosis (38). EMT is critical in various physiological and pathological processes, including tissue fibrosis and cancer metastasis (39). *TWIST1*, similar to *SNAI1*, is a key transcription factor involved in EMT. It has been extensively studied for its roles in embryonic development, cancer metastasis, and fibrosis (38). Both *SNAI1* and *TWIST1* drive EMT by repressing epithelial markers and activating mesenchymal markers, promoting the transition of epithelial cells into mesenchymal-like cells with enhanced migratory and invasive capabilities. These mesenchymal cells secrete cytokines and growth factors, such as transforming growth factor-beta (TGF-β), which activate resident fibroblasts (40). Activated fibroblasts differentiate into myofibroblasts, significantly increasing the production of ECM components, including type I collagen. The gene *COL1A1*, which encodes the alpha-1 chain of type I collagen, is upregulated during this process and contributes to the stabilization and integrity of the ECM. FS is closely associated with ECM remodeling, where changes in the composition and structure of the ECM lead to joint capsule thickening and stiffening, resulting in shoulder pain and restricted movement (41).

*DCN*, a small leucine-rich proteoglycan, interacts with various types of collagen, including type I collagen, and plays a pivotal role in modulating collagen fibrillogenesis, ECM assembly, and tissue repair. *DCN* also has anti-fibrotic properties, inhibiting TGF-β activity (42). Reduced levels or impaired function of *DCN* in FS could lead to unchecked TGF-β activity, increasing collagen production and fibrosis. *TUBB2B*, a gene encoding a member of the beta-tubulin protein family, is essential for the microtubule network within cells (43). Microtubules are essential for the movement of fibroblasts and other cells involved in tissue repair and fibrosis (44). Dysregulation of *TUBB2B* could affect cytoskeletal dynamics, influencing fibroblast migration and invasion into the joint capsule (44), thus contributing to the fibrotic process in FS. In summary, these biomarkers, particularly *SNAI1*, *TWIST1*, *COL1A1*, and *DCN*, are intricately involved in fibrosis, playing significant roles in the development of FS.

ROC curve analysis was performed to assess the diagnostic performance of five genes (*SNAI1*, *TWIST1*, *COL1A1*, *TUBB2B*, and *DCN*), yielding AUC values of 0.845, 0.939, 0.915, 0.915, and 0.842, respectively. These results demonstrate that these genes offer excellent diagnostic accuracy for FS. When these five genes were integrated into a nomogram model, the AUC value increased to 0.970, significantly surpassing the individual gene AUC values. This highlights the nomogram model’s exceptional diagnostic capability, enhancing classification accuracy and reliability. Few studies have addressed the early diagnosis of FS. Xu et al. (35) explored the diagnostic potential of superb microvascular imaging (SMI) features in the rotator cuff gap for FS, identifying SMI blood flow grading as a useful predictor for early FS stages (AUC = 0.824). Moreover, the biomarkers identified in the current study have not been explored in other FS biomarker research. Consequently, the nomogram model could serve as a powerful tool for early FS diagnosis and screening in future clinical applications, improving diagnostic precision and reducing misdiagnosis rates. Further research and clinical trials are necessary to optimize and validate this model for broader clinical use.

The five biomarkers in this study were linked to six main pathways: ribosome, lysosome, drug metabolism-cytochrome P450, ECM receptor interaction, metabolism of xenobiotics by cytochrome P450, and p53 signaling pathway. The ribosome, responsible for protein synthesis, and the lysosome, which serves as the digestive system within cells, are involved in a variety of biological responses and are not specific to FS. The p53 signaling pathway plays a critical role in fibrosis across multiple organs by regulating fibroblast proliferation, ECM synthesis and degradation, oxidative stress, and inflammation, as well as modulating non-coding RNAs (45). Mechanistically, p53 influences fibrosis by upregulating cell cycle inhibitors and pro-apoptotic genes, downregulating ECM synthesis genes, upregulating matrix metalloproteinases and antioxidant genes, inhibiting inflammatory pathways, and regulating miRNA and lncRNA expression (45). These insights offer a theoretical foundation and practical direction for developing new anti-fibrotic therapies.

This study identified 11 immune cells with significantly different infiltration abundances between FS and control samples, all of which were upregulated in FS samples. Immune cell infiltration is crucial in fibrosis, as these cells regulate fibroblast activity and ECM synthesis and degradation through the release of pro-inflammatory or anti-inflammatory factors (46). TGF-β, secreted by immune cells such as macrophages and T cells, activates fibroblasts and promotes excessive collagen synthesis, thereby exacerbating fibrosis (47). Additionally, the inflammatory response is a hallmark of early FS stages. Immune cells like macrophages and T cells release pro-inflammatory substances, leading to localized inflammation and pain. These substances also stimulate fibroblasts, resulting in fibrosis and thickening of the shoulder joint capsule, further limiting joint mobility (48). However, certain immune cells, such as M2 macrophages and regulatory T cells, have anti-inflammatory and pro-repair functions. These cells can inhibit fibrosis progression by secreting anti-inflammatory factors and promoting ECM-degrading enzyme expression (49). Thus, immune cell infiltration plays a dual role in FS pathogenesis, driving both inflammation and fibrosis while possibly aiding tissue repair. Understanding these mechanisms is essential for developing effective therapeutic strategies.

Based on the biomarkers identified, several drugs were predicted to potentially treat FS. Valproic acid may reduce fibrosis by inhibiting histone deacetylase, thus regulating gene expression and decreasing the production of fibrosis-associated proteins (50). Zoledronic acid has been shown to reduce fibrosis by inhibiting fibroblast activity (51). Pamidronate may have anti-fibrotic potential, particularly in bone-related fibrotic diseases (52). Etidronic acid, primarily used to treat osteoporosis and hypercalcemia, may also inhibit certain types of fibrosis (52). However, the efficacy of these drugs for FS treatment requires further validation through animal studies and clinical trials.

In summary, the five biomarkers identified in this study hold significant clinical implications and application potential. For instance, by detecting the expression levels of these biomarkers in patients and comparing them with baseline data from healthy populations, significant discrepancies could assist physicians in more accurately diagnosing FS. Alternatively, monitoring the dynamic changes in the expression levels of these five biomarkers might enable prediction of disease progression trends. Furthermore, for populations with high-risk FS factors - such as family history, specific genetic mutations, or prolonged exposure to relevant environmental factors - regular biomarker testing could facilitate early detection of potential pathological changes before overt symptoms manifest, thereby allowing timely intervention and treatment.

However, there are limitations, including a small sample size that may affect statistical significance and generalizability. Additionally, the lack of systematic animal and cell experiments limits the ability to fully validate the diagnostic and therapeutic value of these biomarkers. Additionally, whether the expression changes of biomarkers and the differences in immune cell infiltration are specific to FS requires further validation. Therefore, we plan to expand the sample size in future analyses to enhance the statistical power and generalizability of the results. Systematic animal experiments will also be conducted to establish FS animal models. By modulating the expression of these biomarkers, we aim to observe their effects on disease progression and immune cell infiltration. Early-stage clinical samples will be analyzed to verify the detectability of these biomarkers in the initial phases of FS. Concurrently, cellular experiments will be performed to explore the mechanisms of interaction between biomarkers and immune cells at the cellular level, validating their diagnostic and therapeutic potential. Furthermore, comparative experiments will be designed, incorporating samples from other shoulder disorders, to clarify the specificity of the identified biomarkers and immune cell infiltration differences in FS.

## 5 Conclusion

This study conducted transcriptome sequencing on FS samples and control samples, identifying five biomarkers through bioinformatics analysis. These biomarkers were found to be involved in pathways such as “drug metabolism-cytochrome P450” and “ECM-receptor interaction,” providing new theoretical references for subsequent in-depth research on FS. Furthermore, we plan to experimentally validate their functions and clinical significance in follow-up studies.

## Data Availability

The datasets presented in this study can be found in online repositories. The names of the repository/repositories and accession number(s) can be found below: PRJNA1192681 (SRA).
