# Supplementary figures and images for: Identification and validation of biomarkers, construction of diagnostic models, and investigation of immunological infiltration characteristics for idiopathic frozen shoulder
Source: Front Immunol. 2025 Jul 16;16:1559422. doi: 10.3389/fimmu.2025.1559422 (PMC12307173; doi:10.3389/fimmu.2025.1559422)

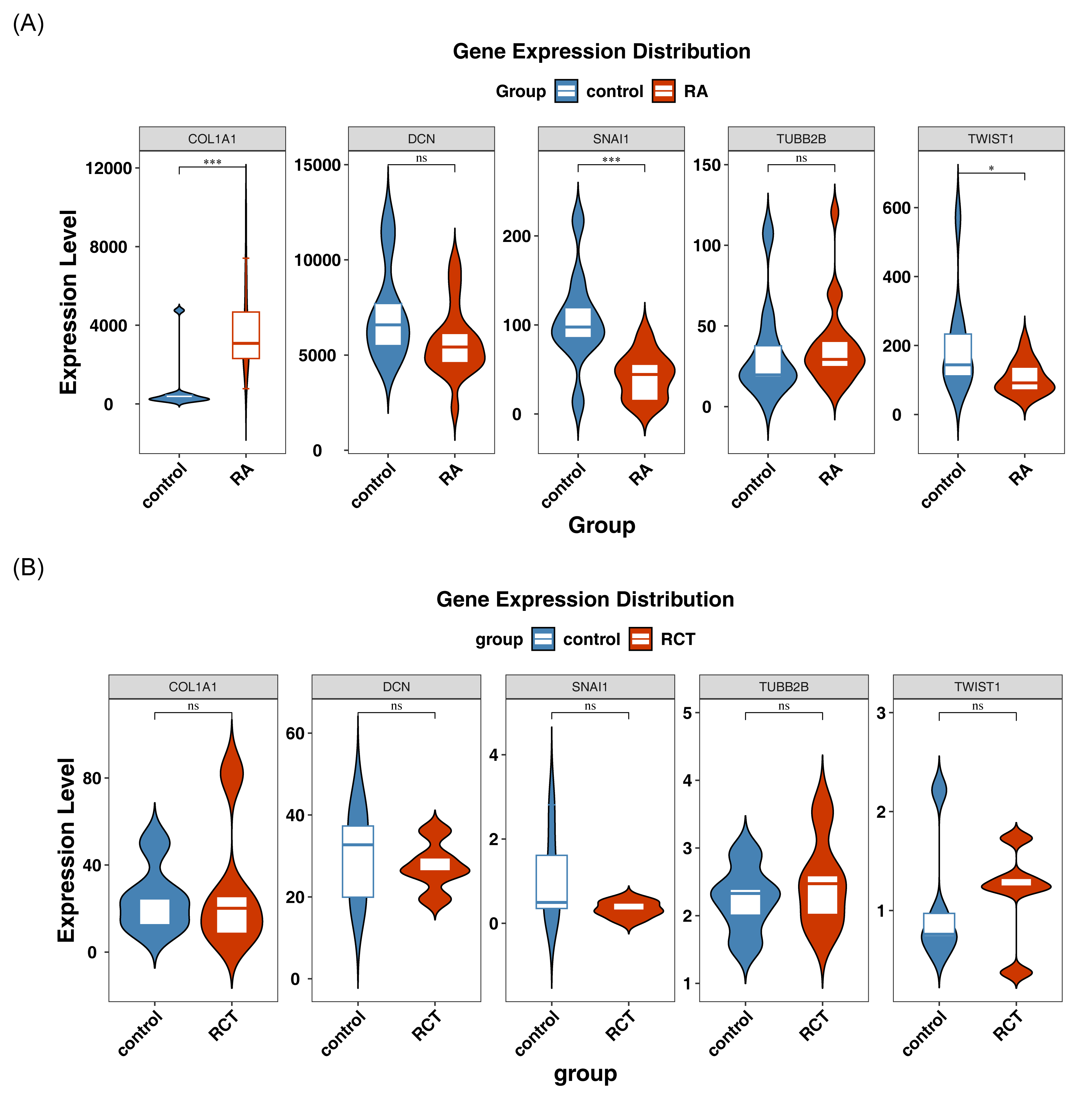

Supplement: Supplementary file 1 [file Image1.tif]
